# Editorial: Interplay between toxicants, natural toxins, and the immune system in animal models

**DOI:** 10.3389/fimmu.2023.1186300

**Published:** 2023-03-31

**Authors:** Mario Bauer

**Affiliations:** Department of Environmental Immunology, Helmholtz Centre for Environmental Research -UFZ, Leipzig, Germany

**Keywords:** immune system, xenobiotic, animal, mitoimmunity, microbiome

An immune system of varying complexity in different species serves both to defend against harmful molecules (toxins) and pathogenic agents that have overcome the host’s natural barriers and to maintain cellular homeostasis by eliminating degenerated and dead cells. Interestingly, the immune system is characterized by great plasticity. By being able to distinguish between self- and non-self-substances, it forms a memory that continues to develop throughout the entire life span. The great challenge for the immune system in the Anthropocene era is to preserve these evolutionarily acquired skills in a permanently changing environment through the release of unnatural anthropogenic xenobiotics and through human activity itself. The risk of adverse effects from single xenobiotics or xenobiotics occurring in a mixture results not only from their released quantity but also from their chemical properties, which contribute to the fact that individual substances and groups of substances are hardly degradable in nature and cannot be metabolized by organisms. This can lead to the lifelong accumulation of these substances, the consequences of which are not yet predictable. To gain a better understanding of the immunomodulatory role of anthropogenic xenobiotics in agriculture, aquaculture, ecology, and conservation, studies on wild and domesticated animals are essential. However, great efforts are still needed to understand the evolutionary range of immunological defense mechanisms against stressors.

One of the substances accumulating in living organisms, as mentioned above, is cadmium (Cd). The intake after occupational or non-occupational exposure to Cd happens unconsciously *via* respiration or ingestion. A physiological function for Cd has not yet been identified, but it poses long-term health risks. Its immunomodulating effects under experimental conditions *in vivo* and *in vitro* in different species has been well summarized in the review by Wang et al. Accumulating also in immune cells, Cd modulates the function of cells of both the innate and adaptive immune systems. The species- and immune cell type-independent adverse effect of Cd on the immune system is mainly immunosuppression with all its consequences. With respect to the half-life time of Cd of decades, the review also emphasized the need to have strategies for the detoxification of living systems from incorporated Cd. Antioxidative bioactive compounds or trace elements, such as zinc or selenium, can be promising candidates for application as supplementation. Although they can prevent the harmful effects of Cd to some extent, they do not promote its excretion. Thus, there is a further urgent need to develop effective immunotherapies to alleviate the immunosuppressing effects of Cd.

Another class of substances of great concern are synthetic brominated flame retardants (BFR) applied to reduce the flammability of products containing them. They are effective in plastics and textile applications such as electronics, clothes, and furniture. They have been detected in animal tissues and in water and sediment samples far from their source of origin, raising concerns about their global impact because of their persistence and bioaccumulation. Baranska et al. described a genotoxic effect of four members of BFR in immune cells, underscoring the immunosuppressing effects of BFR. The compound-dependent genotoxic effect was induced by partially repairable strand-breaks formation and oxidative-damaged pyrimidines in mononuclear cells. This effect was initiated indirectly by DNA-damaging agents, such as reactive oxygen species (ROS) and other reactive species, but not by DNA adducts with BFR. How the genotoxic effect could be prevented was not examined. However, due to the formation of ROS, similar protecting effects by antioxidative compounds, as in the case of damage by Cd, are conceivable.

Apart from the direct immunosuppressing effects of xenobiotics, such as Cd and BFR, an indirect activation of the immune system by a stressor may indicate its detrimental effect. In this respect, Qing et al. have given a convincing example. In pullets, a combination of the two natural feed contaminants, the mycotoxins ochratoxin A (OTA) and aflatoxin B1 (AFB1), evoked pro-inflammatory cytokines in serum as a sign of pathological changes, including enhanced apoptosis in the kidney and liver. Although OTA and AFB1 are known to be toxic to the kidney and hepatotoxic, respectively, the consumption of both toxins additionally influenced the composition of the gut microflora. They stimulated more the growth of pernicious bacteria, from which an additional risk may be posed. Because a permanent interaction between immunocompetent cells and microorganisms in the course of evolution has presumably led to the formation of robust signaling networks that protect the organism, analyzing the effect of xenobiotics or natural toxins on the microbiome is of great importance. If this interaction is disturbed, the immune system is less able to eliminate harmful pathogens.

Immunocompetent cells from germ-free animals show a disturbed cell metabolism and are thus not able to initiate immune responses. Proteins of the mitochondrial respiratory chain seem to play a decisive role in this. Thus, mitochondria represent a key modulator of immune responses (mitoimmunity). Since mitochondria can be particularly vulnerable targets to several classes of environmental toxicants, including metals, polycyclic aromatic hydrocarbons, and pesticides, it can therefore be assumed that these substances can have an immunomodulatory effect by influencing the immune metabolism. As the key role of mitochondria is conserved in vertebrates and *Caenorhabditis elegans*, a free-living nematode, Mello et al. analyzed the effect of the pesticide and inhibitor of the electron transport chain in mitochondria rotenone in *C. elegans* embryos under mild mitochondrial stress. They demonstrated that the mitotoxicant rotenone affected the defense toward individual pathogen species in both directions. The nematodes were more protective against some pathogen species but more unprotected against others.

Due to the diversity of species with their specific immune systems and specific interacting factors (microbiota), it is clear from the outset that this Research Topic can only present examples of studies highlighting the different focuses of the analysis of the immunomodulatory effects of xenobiotics or natural toxins in animals. However, the studies listed above are the most important Research Topics that assess the effects of single stressors and, far more importantly, the mixtures of stressors on the immune system, such as direct and indirect immune modulation, interaction between the immune system and microbiota, and the immune metabolism.

## Author contributions

The author confirms being the sole contributor of this work and has approved it for publication.

